# Hospital and Institutional News

**Published:** 1917-07-14

**Authors:** 


### HOSPITAL AND INSTITUTIONAL NEWS.

### A DIRECTOR OF NEURASTHENIC INSTITUTIONS.

We are officially informed that the Minister of Pensions has appointed Colonel Sir John Collie, R.A.M.C., to be Director of Neurasthenic Institutions. In our issue of June 30 we drew attention to the need for adequate provision and treatment for the increasing number of men incapacitated for military service, at least for the time being, by various forms of nervous disturbance unaccompanied by evidences of organic disease. The problem is important, not only because the sufferers are numerous, but also because, given proper treatment, the great majority of them can be cured—that is, they can be restored either to military or to civil activities. On the other hand, fail to provide this treatment and the patients will continue indefinitely a burden to themselves and to others, and a proportion of them will drift into lunatic asylums. The position thus advances a claim which is economic as well as humane. The appearance of a new Director may be taken as evidence that this claim is receiving adequate attention, and that the steps which have already been taken to meet it are to be efficiently organised. Sir John Collie has had a large experience in dealing both with men and with maladies, and he will bring to his duties important qualifications in the shape of energy and common sense. Doubtless he will be able to secure the co-operation of experts competent to advise both on the neurological and on the psychological aspects of the subject, and thus scientific enlightenment as well as practical results may be achieved.

### AIR-RAID PRECAUTIONS AT GUY'S.

A sheet of concise, but exhaustive, rules signed by the superintendent, Sir Cooper Perry, has been distributed at Guy's Hospital. These rules deal with the conduct of patients and staff in the event of an air-raid, and also with the arrangements for taking in casualties. As the enemy of the greater part of the world has turned to daylight raids as a speciality, and those raids by swiftly moving and sometimes invisible aeroplanes are more deadly than the now old-fashioned Zeppelin excursions, the action of Guy's Hospital is a wise one, and will stimulate similar precautions at other institutions. Confusion is the thing mostly to be feared in these times of emergency, and the rules aim chiefly at preserving order. When the time of danger comes all patients must be brought under cover, and those temporarily absent from their wards must return. All stairs and gangways must be cleared of loiterers, and the lifts are to be reserved for the conveyance of casualties. Important messages will be sent by messenger and not by telephone. A tally label is attached to every patient admitted, on which is written all essential particulars. Stretchers will be returned as quickly as possible to the surgery and stored in the subway. These are but a few of the rules, which cover every conceivable emergency. They illustrate the enormous advantage there is in every member of the staff knowing exactly what is his duty when the moment of danger arrives.

### SIR VICTOR HORSLEY AND COLONEL CARTER.

A remarkable and rather pathetic letter from the late Sir Victor Horsley to Major (now Lieutenant-Colonel) Carter, has come to light. This document (published in full in the *Weekly Dispatch*) was written only eleven days before the great surgeon's death, and is worded in Sir Victor's characteristically straightforward and forcible manner. "Were I the head of affairs," it runs, "I should have made you a Surgeon-General in this war long ago, if only for the further utilisation of your initiative, prescience, and powers of administration. I have seen the ships you equipped for the Indian Government, and I congratulate you on a series of vessels, economical in cost and perfected in every detail, far above the standard hospital ship at home or at Gallipoli. See that as good material is sent out on the river fleet. Men out here are complaining about their instruments and drugs." Had Sir Victor and Colonel Carter had more say in these matters, what a difference it would have made! But Horsley's work was nearly finished; his letter concludes with the words: "Mesopotamia is sapping my strength. I look forward to a change in India." Within a few days he was dead.

### THE RUTHERFORDS AND NEWCASTLE INFIRMARY.

The recent gift by Colonel J. V. W. Rutherford of £1,000 to Newcastle Royal Infirmary, to endow a bed there in memory of his father, is in keeping with the family tradition. Colonel Rutherford's father was actively interested in the old infirmary when it was at Forth Banks, and the colonel himself has completed twenty years' service at the institution. His appointment as honorary consulting surgeon marks his departure from regular active work, but his parting gift will give a new lease of life to his family's association with the hospital's work in the wards. In a letter referring to his retirement Colonel Rutherford described his gift as "a token of his love for the institution."

### THE MOTHER OF CHILDREN'S HOSPITALS.

There is usually a friendly rivalry for titles of this kind, and the activities centring in Baby Week have provided another instance of it. The recent appeal on behalf of the Hospital for Sick Children, Great Ormond Street, was headed by the above title, and now the Liverpool Infirmary for Sick Children, on the occasion of a formal visit by the Lord Mayor of the city, in drawing attention to its own needs, claims the senior position for itself. While wishing success to the appeal of its sister institution, the president and chairman remark that the Hospital for Sick Children in Great Ormond Street was "founded shortly after the establishment of the Liverpool Children's Infirmary." Since the dates can be readily verified, the rival assertions need not detain us. Unlike the hospital in Great Ormond Street, however, the Liverpool Infirmary for Children specifically welcomes new institutions, declares that its own work needs closer co-ordination with them, and that if this involves " an extension of its out-patients' system " the space is available, and the means cannot fail to be found. The Great Ormond Street Hospital complained that among the new proposals it " is practically left to starve," and that it was " better to support " an old and proved institution. Metropolitan and provincial conditions are different, and no doubt largely account for the divergent point of view, but the friendly rivalry remains also a study in different methods of appeal.

### ROYAL INFIRMARY, LIVERPOOL.

The Liverpool Royal Infirmary has for a considerable period undertaken the treatment of venereal diseases. After some delay the proposals of the hospital authorities to bring their practice into line with the scheme of the Royal Commission has been sanctioned by the Local Government Board. The first proposal to renovate and reopen the old Lock Hospital of forty beds, built in 1834 and closed in 1899, was considered to be too costly, inasmuch as the building would be available only for a very limited period. Since 1899 venereal cases have been treated only as out-patients, and last year they numbered 695. It is now intended to add an evening clinic to the days of attendance, and to appoint a staff of assistants for the administration of the department in conformity with the suggestions of the Local Government Board. The hospital has also arranged to admit pre-maternity and maternity cases in connection with the infant-welfare work of the health department of the municipality.

### A DOCTOR'S EDUCATIONAL GARDEN.

The experiment of using a private garden for educational purposes has of recent years been tried at "Westfield," Reading, by Dr. J. B. Hurry. A number of plots has been laid out in which is grown a variety of plants used in industry and commerce. The first series includes plants used in medicine, such as eucalyptus, belladonna, aconite, stramonium, gentian, liquorice, podophyllin, asafœtida, valerian, henbane, castor-oil, cinchona, and the opium poppy. Another series includes plants used for food, like maize, millet, sugar-cane, rice, bananas, arrowroot, ginger, chicory, pepper, olive, and cardamoms. In a third series are shown plants used for clothing and textiles, such as flax, hemp, cotton, jute, phormium tenax, and ramie nettle. There is also a series devoted to plants which yield such dyes as woad, indigo, madder, dyer's weed, turmeric, annatto, and alkanet. Near by in the conservatories are the more delicate economic plants of tea, coffee, soya-beans, monkey nuts, guava, chick pea, cinnamon, and camphor. There is also a small museum in which are collected the various products made from the above-mentioned plants. Each article is accompanied by a descriptive label, so that the living plant can be studied in conjunction with the economic products derived from it. Every summer the garden, conservatories, and museum are thrown open free on several half-holidays to visitors and the older school-children of the borough. A printed catalogue is supplied to every visitor, and from time to time demonstrations of the more interesting exhibits are given by Dr. Hurry and his assistants.

### ONTARIO HOSPITAL, ORPINGTON.

This military hospital, which was built at the cost of the Province of Ontario, and opened in April 1916, began with a complement of 1,040 beds. These were open to all Imperial troops, but the new extension has raised the accommodation to 2,080. Thus doubled in size, the institution is a remarkable tribute to Canadian generosity, and reminds us in the heart of Kent of the solidarity of effort which is being expended on behalf of the troops in every quarter of the Empire. This, too, is well illustrated by the fact that all Imperial troops are eligible for admission to the hospital wards.

### A HOSPITAL CHAIRMAN'S BIRTHDAY.

The Rev. E. Spurrier, chairman of the committee of the Essex County Hospital, has lately attained his eightieth birthday, when he received the congratulations of his colleagues. Mr. Spurrier, who has occupied the position of chairman for the past six years, has had a long and active connection with the hospital, for he has been a member of the committee since 1898. To begin active work on a hospital committee at the age of sixty is not a feat of energy of which most men would be capable. Members of the committee expressed the hope that Mr. Spurrier would be able to preside over the board for many years.

### HIS MAJESTY AS ALMONER.

Major W. Napier Keefer recently placed at the disposal of the King a large money donation, to be applied in such a way as His Majesty deemed most suitable. At the King's request £7,500 of the gift has been allotted by the Joint War Committee of the British Red Cross Society and the Order of St. John as follows: £2,000 to the " Star and Garter " Home for the permanent endowment of a room for paralysed soldiers; £2,000 to the Nurse Cavell Homes of Rest for Nurses, similarly for endowment purposes; £1,000 for the Pinewood Sanatorium for Tuberculous Officers; £1,000 each to the Military Orthopædic Hospital, Dublin, and the Ulster Volunteer Orthopædic Hospital, Belfast; and £500 to the Prisoners of War Central Fund. In addition the King has handed £2,000 to Sir Walter Lawrence, to be used for the benefit of any scheme that may be approved for the treatment of sailors and soldiers suffering from deafness.

### "DOCTOR" AN INTERNATIONAL WORD.

One of the workers of the War Victims Relief Committee of the Society of Friends, describing the work of the Committee in Russia, says: " I still remember the faces of the members of the Zemstvo, how they cleared when we first said 'Doctor' to them. They could not understand the other proposals, but 'Doctor' is the same all the world over. So Lubimofka Hospital was our first-born." She narrates how a tragically empty hospital soon had fourteen beds and an out-patient department busily working. The Relief Committee has now been operating for two and a-half years in France, Belgium, and more recently in Russia. Chiefly from members of the Society of Friends the sum of £136,000 has been raised, and the expenditure has been £114,000. As £1,000 a week is being spent, the balance in hand will last less than six months, and an appeal is issued by Miss A. Ruth Fry, the honorary secretary, at 91 Bishopsgate Street, E.C., for further aid. The helpless in Russia are numbered by millions, and although the Committee cannot hope to do more than touch the fringe of the distress and desolation, they are doing what they can in a field which is absolutely without limit.

### BROOKFIELD RED CROSS HOSPITAL.

A welcome addition has just been completed at the "Brookfield" Red Cross Hospital, Hale End. It consists of an isolation ward of three beds with outside verandah attached for special or serious cases. The addition is the result of the further generosity of Mr. Thomas Armstrong, who last year erected the hospital at his own expense and in his own grounds at Brookfield, Hale End. The patients are also to be congratulated on their good fortune in having recently received through an anoymous donor the gift of the installation of the electrophone in the wards, by which, as is well known, the London churches, theatres, and concert-halls can be brought within sound of those who are lying in bed or are otherwise confined in hospital.

### INFIRMARY MEDICAL SERVICE IN WAR TIME.

Dr. Maurice J. Macauley, assistant medical officer at Bermondsey Infirmary, has accepted a commission in the Royal Army Medical Corps, and the Bermondsey Board of Guardians have decided, with the sanction of the Local Government Board, to pay him half his salary whilst he is with the Forces. The Board are considering a suggestion of Dr. E. S. Bell, the medical superintendent of the infirmary, that Dr. Reid and Dr. Birkett should attend to the out-door poor, who have fallen off considerably in numbers, and that, when either of these doctors is away on weekly leave, an arrangement might be made with some of the local practitioners to attend to urgent cases.

### THE LUCK OF THE MASCOT.

The holding of a Mascot Day has been abandoned. Our readers will remember our description of the proposal, and our chaff of a certain explorer for repudiating the idea of superstition in the same breath as he demanded the return of the original mascot when next he should start on an expedition. We have felt with the public that Flag Days and other days were already overdone, and threatened to become an active nuisance, and therefore we accept the abandonment of Mascot Day with equanimity. But the reason for the withdrawal of the proposal, though not generally published, is interesting. In consequence, we learn, of "rigorous and persistent attacks in some of the so-called 'religious papers,'" the committee in charge of the arrangements has been disbanded. The naturally disappointed secretary believes that as much as £40,000 will be lost to the private hospitals, for whose benefit replicas of Captain Besley's pre-Inca mascot were to be sold. In order, however, that the replicas may not be wasted, and the private hospitals not be deprived of all the benefits expected for them, a letter marked "Mascot," addressed to 1 Marylebone Passage, Wells Street, Oxford Street, enclosing half-a-crown, will secure one of these curiosities, the open sale of which has been prevented.

### THE DONCASTER NEW INFIRMARY.

An important gift has been promised to the new infirmary at Doncaster by the local Co-operative Society. To celebrate the society's jubilee, which will occur next May, the society has decided to provide a new theatre unit at the institution. The unit, the cost of which is not to exceed £3,000, is to bear the society's name, and will comprise the theatre and all its adjuncts. Hospitals rarely benefit from any jubilees except their own, and therefore the promised gift is doubly welcome, and shows a public spirit on the part of the Doncaster and District Co-operative Society on which its members can congratulate themselves.

### A VAST EXTENSION SCHEME.

It is well to emphasise the great step forward which is being taken at Newcastle in connection with the ambitious extension scheme at the Royal Infirmary, Newcastle, full particulars of which we gave in our issue of May 19, page 131. In consequence of the negotiations with the City Council and the Freemen the site of fifteen acres on Castle Leazes will be acquired for the proposed extension as soon as Parliamentary sanction has been gained. That matters would be settled by this arrangement was indicated in The Hospital when the earlier position as regards the site was referred to. The City Council has been friendly and helpful, and the committee proposes to increase the number of beds from 430 to 850. The medical beds would be raised from 177 to 251, and the surgical be doubled and amount to 401. Gynæcological beds would be trebled, and raised to thirty-six; venereal diseases would have twenty-eight beds, and orthopædic cases twenty. This by no means exhausts the proposals, which also include an inevitable extension to the nursing home, a nurses' training-school, a private nursing institution, special quarters for women residents, a students' block, a house governor's residence, and last, but not least, a servants' home and a coroner's court. This scheme, as its next step, requires the promotion of a Bill in Parliament, the cost of which is expected to be £300.

### OFFICERS AND THEIR FOOD.

Lambeth Board of Guardians have reconsidered the request of the officers of their various institutions for a monetary allowance as compensation for the reduction in the value of their rations, by reason of compliance with the regulations of the Food Controller, as reported in The Hospital on June 2, page 167. After a careful examination of the officers' dietary scale, the Guardians are not disposed to make any allowances to them at the present time. They have, however, requested the clerk to draw the attention of the officers to the fact that when they authorised the arrangement of the officers' dietary scale in accordance with the Food Controller's order, permission was given for the purchase of substitutes on the ground that action in accordance with this instruction will prevent any considerable hardship being occasioned by the Guardians' response to the requirements of the Food Controller. They feel that the use of substitutes is somewhat difficult, but realise that in this respect the officers would be in no less advantageous a position than the ordinary householder.

### THE ABRAM PEEL HOSPITAL.

Accommodation for a further 350 wounded men has been provided in Bradford by the conversion of the Fever Hospital to their use. The hospital has been re-christened the Abram Peel War Hospital, by the Health Committee of the Bradford Corporation, in acknowledgment of the services rendered by the Lord Mayor to the sick and wounded. The hospital was recently inspected by Surgeon-General Bedford, C.B., the Deputy-Director of Medical Services for the Northern Command, and formally handed over to him by the Lord Mayor. In his speech General Bedford explained that the hospital would be affiliated to St. Luke's War Hospital, though its finances would be distinct. The West Riding, he added, had not only given thousands of men to the Army, but a number of hospitals also. Mr. E. J. Smith, chairman of the Health Committee, deserves great credit for the way in which the hospital has been refitted. The medical officer is Dr. Kitchen, and the matron Miss Lewis.

### MENINGITIS IN NEW YORK.

A special division of the New York Health Department was established in 1910 for the purpose of combating epidemic cerebro-spinal meningitis, which is very prevalent in New York and many other eastern cities of the United States. The division is intended to assist physicians in the diagnosis and treatment of cases of this disease, but patients are seen only in consultation with the attending practitioner, and this is done for several reasons; there is no wish to infringe on the province of the attending physician, and it is much easier to get the consent of the parents to a lumbar puncture if their own doctor tells them that it is necessary. The division deals with all forms of meningitis, and the first thing done if the symptoms show that some form of meningitis is present is to make a lumbar puncture, and the cerebro-spinal fluid is then examined. If there is any reason for thinking from this examination that the case is one of epidemic meningitis, a dose of serum is administered. If the case proves to be not epidemic meningitis, no harm will have been done; while if it is, valuable time will have been saved in treatment. In epidemic meningitis it is rarely wise to give less than four injections on consecutive days, and it may be necessary to give many more. The injections must be continued until the fluid has become sterile and the symptoms have greatly improved. For the relief of pressure, lumbar puncture is important, in addition to its value for diagnosis. With regard to the result of the establishment of this division, it is difficult to give any exact figures as to its value, but certainly the number of cases seen has greatly increased in the five years of its existence. In 1911, 122 cases were seen, but in 1915 the number had risen to 246. The mortality of the disease still continues to be very high, for even in the last year it was 34 per cent., but it must be remembered that many of the cases are seen after they have been ill a fortnight or more. It is clear that the division is doing good work, but more will be possible when the mystery of the mode of transmission of the disease has been cleared up.

### CORONER'S TRIBUTE TO ST. BARTHOLOMEW'S HOSPITAL.

Dr. F. J. Waldo, Coroner for the City of London, has written to the clerk of St. Bartholomew's Hospital in connection with the air raids in London, stating that at the recent inquiries held by him in the City Court concerning the deaths of the numerous victims, the juries added unanimous riders to their verdicts placing on record their high appreciation of the most valuable services rendered by the doctors, nurses, and staff generally, attached to the hospital. Dr. Waldo adds: "My officers have informed me of the splendid manner in which the sisters and nurses carried out their arduous duties in the surgery on the day of the raid. I would like to take this opportunity of heartily endorsing every word recorded by the juries, and of offering my congratulations to the various members of the staff of St. Bartholomew's Hospital."

### AN ELECTRICAL INSTALLATION FOR MANSFIELD.

The Duke of Portland has offered an electrical installation for the treatment of paralysis, rheumatism, and other suitable maladies to the Mansfield and District Hospital, on condition that the funds for its maintenance are provided. The governors are taking steps to comply with the condition. His Grace has also given a hundred patent pneumatic crutches to the Nottingham Hospital. These crutches are the invention of the Duke's motor engineer, Mr. Hunter, and are being manufactured in Mansfield. The resilient cushion at the head of the crutch distributes the weight of the user better than the old wooden arm, and reduces crutch paralysis to a minimum.

### GERMAN FEEDING-CUPS FOR THE RED CROSS.

It appears that we have not even yet wholly got rid of German goods. In answer to a question put by Mr. R. Gwynne in the House of Commons, Mr. Macpherson, the Under-Secretary for War, had to admit that some of the feeding-cups supplied to

V.A.D. hospitals by the Red Cross were of German manufacture, and had attached to them a label bearing the words "Krankenbecher, 665, weiss," which means a beaker for the sick, white in colour. The explanation given by Mr. Macpherson for the use of these cups was that at the beginning of the war, owing to the abnormal demand, it was necessary to secure all available stocks of enamel ware. The stocks were bought from English firms, but as little or no enamel ware of this nature was made in England, the original source of supply was no doubt foreign. He deprecated, however, the suggestion that money subscribed by the British Red Cross was going to Germany in payment for these cups. When Mr. Gwynne asked if he was to infer from Mr. Macpherson's statement that we were still using feeding-cups bought at the commencement of the war, the answer was in the affirmative.

### THE PALAIS DE GLACE.

The *Times* gives a brief account of a hospital in Paris, which has been called the Palais de Glace, a name which may be translated either the ice palace or the glass palace. In its construction several devices have been employed which are not used extensively, so far as we are aware, in any other hospital. The Palais de Glace is a children's hospital; it was founded by Dr. Lesage, and he is still the visiting physician. The first noticeable point about the hospital is that the methods of ventilation employed in it differ much from those adopted in most other hospitals. By means of ever-open doors and perforated window panes there is a provision of fresh air to the wards, and yet we are told that there is a complete absence of draughts. In the second place every patient is isolated in a cubicle, but the walls of the cubicle are composed entirely of glass, so that children in neighbouring cubicles can see and, it is said, talk to one another without any risk of the communication of disease. Therefore, we are told, it has been found perfectly possible and safe to have in the same ward cases of measles, scarlet fever, typhoid, and pneumonia, and to treat them side by side without any fear or risk of infection. The details of the arrangements in this hospital given in the report are not sufficient to enable a judgment to be formed as to their suitability, and any considered expression of opinion would be impossible; but it may be said that the use of glass partitions is not entirely new, and this material certainly has advantages in cleanliness and durability; its cost is somewhat against it, for the partitions must be thick to minimise the risk of breaking. Further, the use of perforated glass for ventilation has often been employed, but the prevention of a draught is by no means easy, as all will agree who have had much experience in the ventilation of the wards of a hospital.

### ASHTON INFIRMARY AND THE EXPLOSION.

In respect of destruction of life and property, the terrific explosion at Ashton-under-Lyne on June 13 seems the counterpart of the catastrophe at Silvertown in January last. The effects were felt two miles away at the district infirmary, while several windows were broken by the shock. Although many patients were naturally upset there was no panic; and, without waiting for any police warning—it being apparent that an accident on a large scale had taken place—arrangements were immediately made to receive casualties. The patients' waiting-hall was cleared of benches and filled with bedsteads, and the whole of the available staff, including the night nurses, stood by for duty. In a very few minutes a stream of wounded began to arrive in vehicles of all descriptions, and scenes, fortunately without parallel in the history of the infirmary, ensued; men, women, and children, some beyond human aid, some fortunately more frightened than hurt, were hurried in. In all, thirty-five severe operations were performed in the two operating theatres, while the minor casualties were being attended to in the casualty reception room. On the first night fifty-eight beds were occupied by the victims of the explosion.

### ASHTON HOSPITAL WORKERS' THANKS.

Many acts of heroism in connection with the explosion at Ashton are recorded by the local press. The *Reporter* describes the heroic death of Mr. Sylvain Drefus, the manager of the destroyed factory, who, having, like Dr. Angel at Silvertown, done his utmost to secure the safety of his fellow-workers, perished at the post of duty. A well-deserved tribute is paid to the untiring efforts of the staff of the Ashton Infirmary. It is clear that the honorary medical staff, the house surgeons, the matron and nurses, and all concerned rose magnificently to the sudden emergency. Equal praise must be accorded to Red Cross nurses and V.A.D. workers, the authorities of the Poor-Law Institution, and the Ryecroft Hall and Richmond military hospitals, and many others. Miss Katherine F. Cooper, the matron of the infirmary, has publicly expressed her thanks and gratitude to all those (particularly the nurses and V.A.D.s) who volunteered their services, and Miss Emily Price, the lady superintendent of No. 236 Women's V.A.D., East Lancashire, has similarly thanked her members and the men of No. 47 British Red Cross Detachment, for assistance to the injured and suffering at the hospital, on the scene of the disaster and in various parts of the town. It must soften to some degree the public distress caused by the explosion to know that undoubtedly many lives were saved by the immediate attention given to the sufferers.

### GOATS FOR INSTITUTION MILK.

A shortage of milk next winter is anticipated, and some authorities have ordered stocks of dried milk. At Lambeth the Board of Guardians have purchased seven goats, which cost in all £23 10s. Good milkers, it was reported, are very expensive at the present time, and animals of this class could not be purchased, the price asked ranging from £8 to £12 each. The seven which the Board has bought are young, and, it is hoped, will develop into good milkers next season. The Board, by the way, has also bought some young rabbits at 1s.

each for the purpose of fattening. These can be reared cheaply on the pasture of the Poor-Law School, and may, perhaps, prove a profitable investment.

### SPHAGNUM MOSS.

A new industry is now being carried on among the extensive peat marshes and on the banks of the innumerable rivulets of Dartmoor. Sphagnum moss has recently acquired an especial value in connection with war work. Bands of volunteer war workers scour the rugged tors and the windswept valleys lying between them, and, whatever the weather may be, they search for the precious moss. One of the most active workers has succeeded in gathering during the past two months over 200 sackfuls. The crop is taken to premises at Princetown placed at the disposal of the Queen Mary's Needlework Guild by the Prince of Wales, who also bore the cost of fitting the building for its purpose. Princetown is the little capital of Dartmoor, and it is here that the great convict prison—now, however, given over to the conscientious objectors—is situated. Under the direction of the prison governor's wife, the workers at the depôt (most of them the wives of warders) carry out the cleansing of the moss. This is tedious and tiresome work, for a species of grass which clings to the moss like a parasite has to be carefully separated from it. Having been picked clean, the soaking sphagnum is placed on wire-netting trestles to be dried by heat and currents of air. Then it is sewn up in muslin bags, so that the weight of each bag when full is exactly 2½ oz. These "pillows"—as they are called—are dipped for a moment in a mercurial emulsion, and then squeezed until their weight is just 4 oz. When sphagnum moss was first used for surgical dressings the process of sterilisation by heat was adopted, but this method, it has been found, tends to destroy the absorbent qualities of the moss, so the newer process of "sublimation" recommended by Colonel Charles Cathcart, F.R.C.S., the pioneer in the use of sphagnum for dressings, is now generally followed. After sublimation the pillows are hung in a drying chamber with a temperature of not less than 90 degrees for twenty-four hours. At the end of that time the moss is proof against the reception of any form of bacteria, and its absorbent capacity is such that it can take up eighteen times its own weight of moisture. Finally, the pillows are packed in sets of five in sheets of paper. Bundles of ten are then wrapped together in waterproof sheetings and despatched in airtight hampers (made at a neighbouring blind institution) to London, whence they are distributed to the dressing hospitals, especially those at the base.

### AN INFIRMARY FIRE.

Recently there was a fire at the children's infirmary of the Lambeth Board of Guardians. It did not attain to any large dimensions, but this was evidently due to the prompt action of Nurse Fisher, for, in sending the Board a cheque for £3 19s. for the damage, the County Fire Office enclosed half a guinea for the nurse in recognition of the prompt manner in which she dealt with the outbreak.

### THE SALARIES OF MEDICAL WOMEN.

The medical woman is coming to her own, as is shown by incidents in Bethnal Green and Stoke-on-Trent. The Bethnal Green Borough Council advertised for a medical woman to act as assistant medical officer of health primarily for the purpose of the proposed welfare centre, offering £350 a year. There were only two replies, and only one was prepared to accept the salary offered, and she proved unsuitable. Now the Council proposes to advertise again, with the inducement of a salary of £400 a year. Stoke-on-Trent County Borough Council wished to advertise for an assistant medical woman at a salary below the £350 minimum stipulated by the British Medical Association, but found that the advertisements were banned. Now it has been decided to offer that £350 minimum instead of £300 as originally proposed. Moreover, the Council has increased the salary of the present woman medical officer, Dr. Balsillie, from £350 to £400 a year.

### THIS WEEK'S DRUG MARKET.

A question that is being discussed in drug trade circles is whether the Government are likely to extend their control of the drug market. At present very few articles are under direct Government control, but experience of the result of the Government control of quinine suggests that hospitals using large quantities of drugs might benefit considerably if various other commonly-used drugs were put in the same category as quinine. For instance, stocks of quinine have been reduced substantially without any noteworthy alteration in the price of the drug, but there is no reason to suppose that the price would have remained stationary had there been no control of the drug. Generally speaking the drug market is quiet, and the upward tendency of prices is maintained. Salicylic acid and sodium salicylate are again dearer, and in view of the large quantities of these drugs required by the medical services of our own and our Allies' armies, and the practically unabated demand among the civilian population, it would not be surprising if prices advanced still higher, for the output is inadequate. Atropine is rather dearer. Remarkable prices are asked for any permanganate of potash available. Other drugs that have advanced in price include silver nitrate, tartarated antimony, hypophosphites, diacetyl hydrochloride of morphine, morphine ethyl hydrochloride, and thymol. There is an upward tendency in the prices of rhubarb, gum benzoin, and eucalyptus oil.

### TO OUR READERS.

**Contributions are specially invited from any of our readers to these columns. They should deal with topical subjects and news. They must be authenticated for the information of the Editor only. The minimum payment if published is 5s. There is no hard-and-fast rule as to space, but notes of about twenty lines in length are preferred.**

